# Similar intraoperative microbiomes in *de novo* and replacement cardiac implantable electronic device pockets support transient contamination over stable colonisation

**DOI:** 10.3389/fcimb.2026.1843081

**Published:** 2026-07-06

**Authors:** Zhe Xu, Xuehai Chen, Kezeng Gong, Feilong Zhang, Yunling Lin

**Affiliations:** 1 Department of Cardiology, Fujian Medical University Union Hospital, Fuzhou, China; 2 Fujian Cardiovascular Medical Centre, Fuzhou, China; 3 Fujian Institute of Coronary Heart Disease, Fuzhou, China; 4 Fujian Cardiovascular Research Centre, Fuzhou, China; 5 Fujian Medical University Heart Centre, Fuzhou, China

**Keywords:** 16S rRNA sequencing, aseptic technique, contamination, healthcare-associated infection, pacemaker, surgical site infection

## Abstract

**Background:**

The source of bacteria detected in cardiac implantable electronic device (CIED) pockets—whether true colonisation or intraoperative contamination—has significant implications for infection prevention strategies.

**Aim:**

To compare intraoperative microbial dynamics during *de novo* pacemaker implantation versus generator replacement using 16S rRNA gene sequencing.

**Methods:**

In this prospective study, samples were collected from 18 asymptomatic patients (11 new implants, seven replacements) at three timepoints: skin before incision (T1), pocket after creation (T2), and pocket before closure (T3). Microbiome analysis was performed via 16S rRNA gene sequencing of the V3-V4 region. α-diversity (Simpson index) and β-diversity (Bray–Curtis dissimilarity) were analysed.

**Findings:**

Microbial community composition and relative abundance were similar between groups at all timepoints. Predominant genera included A*chromobacter, Pedobacter, Pseudomonas, Acinetobacter, Blastococcus, and Paracoccus*. No significant differences in α- or β-diversity were found between new and replacement groups at T2 and T3. The only significant difference was a higher α-diversity at T1 (skin) in the new implantation group (*P* = 0.037), likely reflecting age-related skin flora differences. No patient developed a clinical infection during follow-up (mean 9.4 ± 1.8 months).

**Conclusion:**

The absence of distinct microbial signatures in replacement pockets and the prevalence of environmental bacteria strongly suggest that detected microorganisms originate from intraoperative contamination. This underscores the paramount importance of rigorous aseptic technique over surveillance for colonization in preventing CIED infections.

## 1 Introduction

Infections associated with cardiac implantable electronic devices (CIEDs) are serious healthcare-associated events, leading to significant morbidity, mortality, and economic cost (Didier et al., 2010). A hypothesised precursor to clinical infection is subclinical bacterial colonisation within the device pocket (Blaser and Kirschner, 2007; Rohacek et al., 2010). Studies using culture-based methods have reported positive swabs or tissue cultures in up to 40% of asymptomatic patients undergoing device revision, with Cutibacterium spp. predominating (Pichlmaier et al., 2008; Mason et al., 2011). However, these findings are difficult to interpret due to the inherent risk of contamination during surgery or sample processing.

If true colonisation exists, one would expect a higher bacterial load and a distinct microbial community in patients with long-term implants (replacement group) compared to those receiving a first device (new implantation group). Culture-based studies have not consistently supported this hypothesis (Okada et al., 2015). The inability of culture to distinguish colonising pathogens from contaminants complicates clinical decision-making and may lead to overtreatment.

Advances in molecular techniques, such as 16S rRNA gene sequencing, allow for a more comprehensive and culture-independent analysis of microbial communities. This prospective study utilised microbiome analysis at defined intraoperative timepoints to characterise the origin of bacteria detected during CIED surgery. Clarifying whether microbes arise from contamination or pre-existing colonisation is essential for directing effective infection prevention and control (IPC) resources, such as optimising aseptic technique versus implementing surveillance culturing.

## 2 Methods

### 2.1 Study design and patients

This prospective study enrolled patients undergoing *de novo* pacemaker implantation or generator replacement between June and December 2022 at Fujian Medical University Union Hospital. Patients with clinical evidence of active infection, immunosuppression, acute heart failure, renal insufficiency (estimated glomerular filtration rate < 30 mL/min/1.73 m²), or those requiring implantable cardioverter-defibrillator/cardiac resynchronisation therapy device procedures were excluded. The study protocol was approved by the hospital Ethics Committee (Approval No. 2019JYKY008). Written informed consent was obtained from all participants.

### 2.2 Surgical procedure and sample collection

All procedures were performed in a catheterisation laboratory meeting Grade II clean operating room standards (GB 50333-2013). Standard skin antisepsis was performed with 7.5% povidone–iodine. Prophylactic antibiotics (cefazolin or clindamycin) were administered intravenously 30–60 min before incision.

For each patient, three samples were collected using sterile saline-soaked gauze: (T1) skin at the planned incision site before incision; (T2) inside the device pocket immediately after its creation; (T3) inside the pocket before wound closure. Sampling times were recorded to calculate ‘pocket creation time’ (T1 to T2) and ‘device operation time’ (T2 to T3).

### 2.3 Microbiome analysis

Genomic DNA was extracted using the FastPure Stool DNA Isolation Kit(Magnetic bead) (MJYH, shanghai, China) according to the manufacturer’s instructions. This extraction workflow had been routinely used in our laboratory for bacterial 16S rRNA sequencing analyses and consistently generated sufficient DNA for successful amplification and downstream sequencing of all study specimens. The V3-V4 region of the bacterial 16S rRNA gene was amplified with primers 341F/805R and sequenced on an Illumina MiSeq platform (2×300 bp). Negative controls (sterile saline-soaked gauze) were processed concurrently. Sequences were processed using Mothur v1.30.2 and QIIME v1.9.1, with taxonomy assigned against the SILVA 138 database. Samples with >0.1% reads matching negative controls were excluded. α-diversity was assessed using the Simpson index. β-diversity was calculated using Bray–Curtis dissimilarity and visualised via principal co-ordinate analysis.

### 2.4 Clinical follow-up

Patients were followed up at 1, 3, 6, and 12 months postoperatively to monitor for signs of pocket infection or systemic infection according to modified Duke criteria.

### 2.5 Statistical analysis

Continuous variables are presented as mean ± standard deviation and compared using Student’s *t*-test or Mann–Whitney U-test. Categorical variables are presented as numbers (%) and compared using the χ² test or Fisher’s exact test. The Simpson index was compared using the Wilcoxon rank-sum test. Differences in β-diversity were assessed using analysis of similarities (ANOSIM). A P value < 0.05 was considered significant. Analyses were performed with SPSS v20.0.

## 3 Results

### 3.1 Patient characteristics

Eighteen patients were enrolled (11 new implants, seven replacements). Baseline characteristics are shown in **Table 1**. Patients in the replacement group were significantly older and had a higher body mass index. The device operation time was significantly shorter in the replacement group, as expected.

**Table 1:** Baseline characteristics of the study patients.

Variable	Total (18)	New (11)	Replacement (7)	*P*
Male, n (%)	5 (27.8)	2 (18.2)	3 (42.9)	0.326
Age, years	65.38 ± 11.47	60.54 ± 10.93	73.00 ± 7.95	0.020
Body mass index, kg/m^2^	23.95 ± 2.89	22.58 ± 2.10	25.72 ± 2.92	0.025
Serum albumin, g/L	38.29 ± 3.49	37.27 ± 3.74	39.75 ± 2.71	0.155
Baseline disease				
Hypertension, n (%)	9 (50)	4 (36.4)	5 (71.4)	0.335
Diabetes, n (%)	3 (16.7)	1 (9.1)	2 (28.6)	0.528
Prosthetic heart valve, n (%)	2 (11.1)	2 (18.2)	0 (0)	0.497
Medications				
Anticoagulant, n (%)	4 (22.2)	4 (36.4)	0 (0)	0.119
Antiplatelet, n (%)	3 (16.7)	1 (9.1)	2 (28.6)	0.528
Procedure				
Pocket creation time, min	16.55 ± 9.12	17.09 ± 5.28	15.71 ± 13.71	0.765
Device and lead operation time, min	21.16 ± 16.31	31.72 ± 12.19	5.71 ± 4.9	< 0.0001

### 3.2 Microbiota composition and diversity

**Figure 1:**
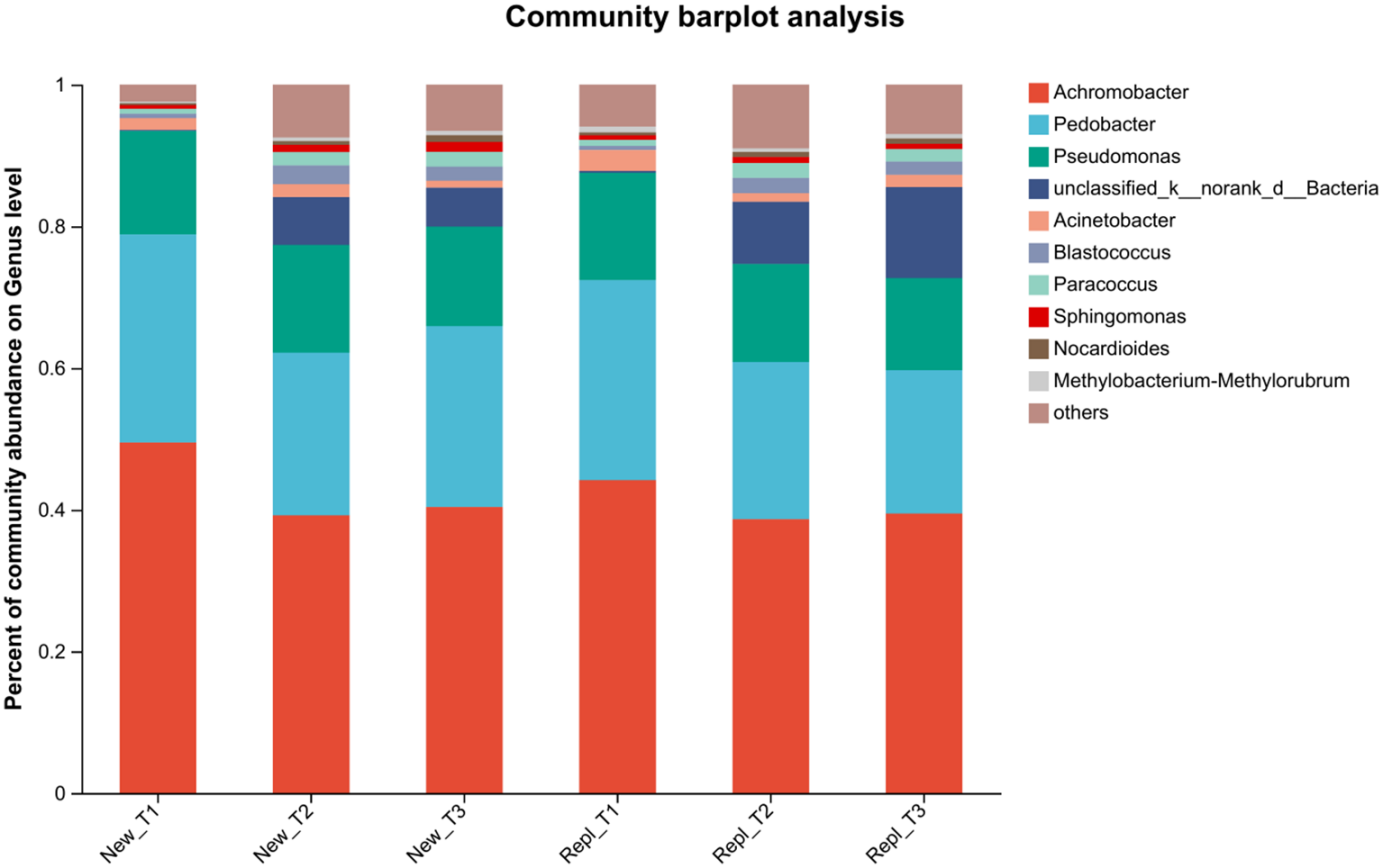
Bacterial community composition at the genus level for the new implantation group (New) and the replacement group (Repl) at three intraoperative timepoints: T1 (skin before incision), T2 (pocket after creation), T3 (pocket before closure). Stacked bars show the relative abundance of the predominant genera.

Bacterial DNA was detected in all samples. The predominant genera were *Achromobacter, Pedobacter, Pseudomonas, Acinetobacter, Blastococcus, and Paracoccus*. Community composition and relative abundance were similar between the new and replacement groups at each corresponding timepoint (T1, T2, T3) (**Figure 1**).

**Figure 2:**
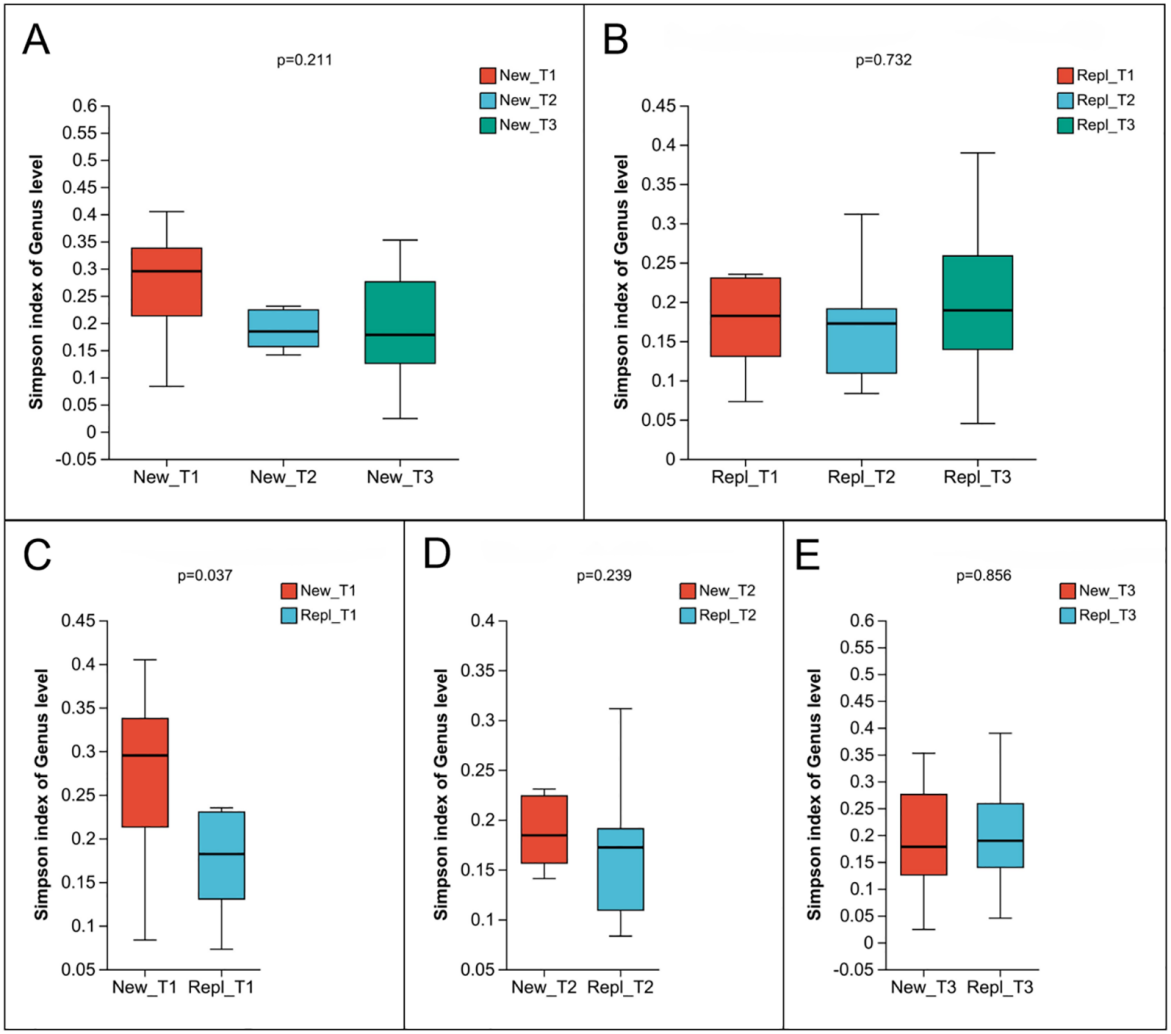
α-diversity (Simpson index) analysis. **(A)** Comparison across timepoints within the new implantation group. **(B)** Comparison across timepoints within the replacement group. **(C–E)** Comparison between the new and replacement groups at T1 **(C)**, T2 **(D)**, and T3 **(E)**.

No significant differences in α-diversity (Simpson index) were observed within each group across the three timepoints (**Figures 2A, B**). At T1 (skin), the new implantation group had a significantly higher α-diversity than the replacement group *P* = 0.037 (**Figure 2C**). However, no significant differences in α-diversity were found between the two groups at T2 (after pocket creation) or T3 (before closure) (**Figures 2D, E**).

**Figure 3:**
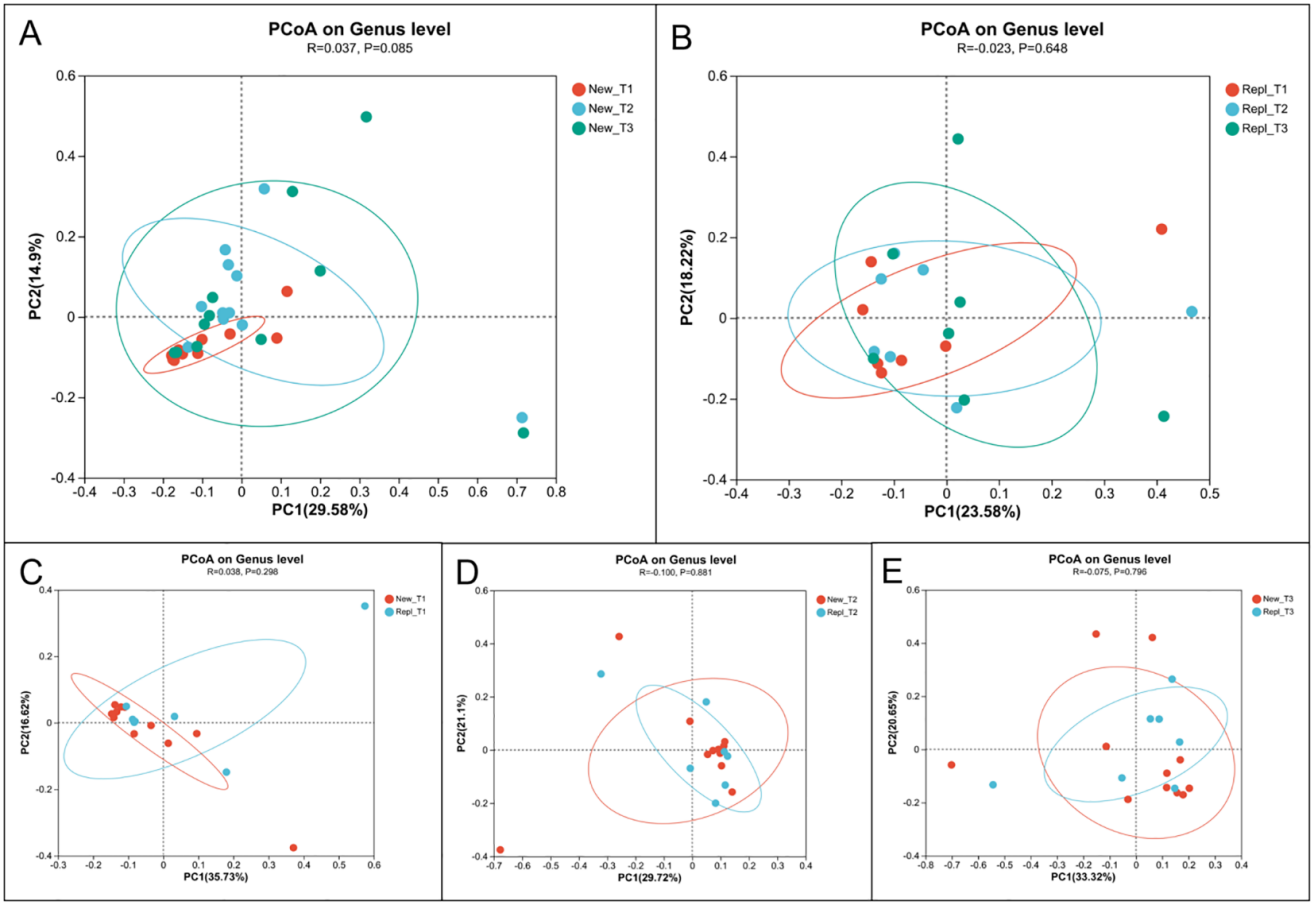
β-diversity analysis based on Bray–Curtis dissimilarity. **(A)** Principal coordinate analysis (PCoA) of samples from the new implantation group across three timepoints (T1, T2, T3). **(B)** PCoA of samples from the replacement group across three timepoints. **(C–E)** Comparison of β-diversity between the new and replacement groups at T1 **(C)**, T2 **(D)**, and T3 **(E)**. Ellipses represent 95% confidence intervals for each group. Statistical significance was assessed using ANOSIM.

Analysis of β-diversity (Bray–Curtis dissimilarity) revealed no significant differences within each group across timepoints (ANOSIM *P* > 0.05) or between the new and replacement groups at any timepoint (T1, T2, T3) (ANOSIM *P* > 0.05) (**Figure 3**).

### 3.3 Clinical outcomes

During a mean follow-up of 9.4 ± 1.8 months, no patient developed a CIED-related pocket infection or systemic infection.

## 4 Discussion

This prospective study utilised serial intraoperative sampling and 16S rRNA gene sequencing to trace the origin of bacteria in CIED pockets. The key finding is the lack of a distinct microbial signature in replacement pockets compared to newly created pockets. The similar diversity and composition at T2 and T3, dominated by environmental genera such as *Achromobacter* and *Pseudomonas*, strongly implicate intraoperative contamination as the primary source.

Our findings challenge the paradigm that positive microbiological findings in asymptomatic patients primarily represent significant colonisation (Pichlmaier et al., 2008; Mason et al., 2011). Instead, they align with and extend the work of Okada et al., who, using culture, found similar contamination rates in new and replacement procedures (Okada et al., 2015). Molecular techniques reveal a broader spectrum of contaminants, highlighting the limitation of culture which may over-represent skin commensals like *Cutibacterium*. Our findings are broadly consistent with previous studies of asymptomatic CIED pockets, which have predominantly identified low-virulence skin-associated microorganisms rather than classic invasive pathogens (Okada et al., 2015). Differences in study populations, sampling strategies, specimen processing methods, microbiological detection techniques, and analytical workflows may nevertheless contribute to variations in microbial profiles reported across studies.

From an IPC perspective, these results are reassuring. They suggest that the low-biomass microbial ingress occurring during even routine, aseptic surgery is typically cleared by host defences and perioperative antibiotic prophylaxis, explaining the low incidence of overt infection in our cohort and others (Kleemann et al., 2010). This understanding should steer clinical focus away from potentially unnecessary interventions aimed at eradicating presumed ‘colonisation’ in asymptomatic individuals.

The significant difference in skin (T1) microbiota diversity between younger (new implant) and older (replacement) patients is consistent with known age-related changes in skin flora (Somerville, 1969; Rafat et al., 2017), but this difference was erased once the pocket was opened, further underscoring the shared contamination pathway during surgery.

The primary implication for IPC practice is the reinforced critical importance of meticulous aseptic technique, optimal skin antisepsis, controlled operating room environments, and strict adherence to surgical checklists. Efforts to reduce CIED infections should prioritise these fundamental measures over surveillance culturing in asymptomatic patients.

## 5 Limitations

This study has limitations. The sample size is small, which may limit the power to detect subtle differences. The follow-up period, while exceeding nine months, may not capture very late-onset infections. Although we employed stringent negative controls, the extreme sensitivity of sequencing cannot completely rule out minor reagent or environmental background signal. As with all low-biomass microbiome studies, DNA extraction efficiency may influence recovery of low-abundance taxa (Gall-David et al., 2023). Consequently, microorganisms present at very low abundance may remain below the detection threshold of sequencing-based approaches. However, all specimens were processed using an identical extraction and sequencing workflow, reducing the likelihood that methodological bias affected comparisons between study groups. Larger, multicentre studies with longer follow-up are warranted to confirm these findings.

## 6 Conclusion

Microbiome analysis of samples collected during CIED surgery indicates that detected bacteria are likely introduced via intraoperative contamination rather than representing pre-existing colonisation. This finding underscores that strengthening foundational infection prevention measures, such as aseptic technique and operating room discipline, remains the cornerstone of preventing device-related infections.

## Data Availability

The datasets presented in this study can be found in online repositories. The names of the repository/repositories and accession number(s) can be found below: https://www.ncbi.nlm.nih.gov/, PRJNA1440190.

## References

[B1] Blaser M. J. Kirschner D. (2007). The equilibria that allow bacterial persistence in human hosts. Nature 449, 843–849. doi: 10.1038/nature06198 17943121

[B2] Didier K. Frederic W. Salem K. Courcol R. (2010). Positive cultures in asymptomatic patients during elective device replacement: a murderer hides in the darkness or an innocent person on the crime scene? Europace 1, 5–6. doi: 10.1093/europace/eup402 20008492

[B3] Gall-David S. L. Boudry G. Buffet-Bataillon S. (2023). Comparison of four DNA extraction kits efficiency for 16SrDNA microbiota profiling of diverse human samples. Future Sci. OA 9, FSO837. doi: 10.2144/fsoa-2022-0072 37006230 PMC10051199

[B4] Kleemann T. Becker T. Strauss M. Dyck N. Weisse U. Saggau W. . (2010). Prevalence of bacterial colonization of generator pockets in implantable cardioverter defibrillator patients without signs of infection undergoing generator replacement or lead revision. Europace 12, 58–63. doi: 10.1093/europace/eup334 19861383

[B5] Mason P. K. Dimarco J. P. Ferguson J. D. Mahapatra S. Mangrum J. M. Bilchick K. C. . (2011). Sonication of explanted cardiac rhythm management devices for the diagnosis of pocket infections and asymptomatic bacterial colonisation. Pacing Clin. Electrophysiol. 34, 143–149. doi: 10.1111/j.1540-8159.2010.02820.x 20561226 PMC4555211

[B6] Okada M. Kashiwase K. Hirata A. Nemoto T. Matsuo K. Murakami A. . (2015). Bacterial contamination during pacemaker implantation is common and does not always result in infection. Circ. J. 79, 1712–1718. doi: 10.1253/circj.cj-15-0133 25971526

[B7] Pichlmaier M. Marwitz V. Kühn C. Niehaus M. Klein G. Bara C. . (2008). High prevalence of asymptomatic bacterial colonization of rhythm management devices. Europace 10, 1067–1072. doi: 10.1093/europace/eun191 18658253

[B8] Rafat Z. Hashemi S. J. Ahamdikia K. Daie Ghazvini R. Bazvandi F. (2017). Study of skin and nail Candida species as a normal flora based on age groups in healthy persons in Tehran-Iran. J. Mycol. Med. 27, 501–505. doi: 10.15406/bbij.2019.08.00269 28967539

[B9] Rohacek M. Weisser M. Kobza R. Schoenenberger A. W. Pfyffer G. E. Frei R. . (2010). Bacterial colonization and infection of electrophysiological cardiac devices detected with sonication and swab culture. Circulation 121, 1691–1697. doi: 10.1161/circulationaha.109.906461 20368521

[B10] Somerville D. A. (1969). The normal flora of the skin in different age groups. Br. J. Dermatol. 81, 248–258. doi: 10.1111/j.1365-2133.1969.tb13976.x 5778713

